# A Systematic Review and Meta-Analysis on Opioid Management of Dyspnea in Cancer Patients

**DOI:** 10.3390/cancers17081368

**Published:** 2025-04-19

**Authors:** Elena Chitoran, Vlad Rotaru, Giuseppe Gullo, Daniela Viorica Mosoiu, Laurentiu Simion

**Affiliations:** 1 Medicine School, “Carol Davila” University of Medicine and Pharmacy, 050474 Bucharest, Romania; 2 General Surgery and Surgical Oncology Department I, Bucharest Institute of Oncology “Prof. Dr. Alexandru Trestioreanu”, 022328 Bucharest, Romania; 3 Department of Obstetrics and Gynecology, Villa Sofia Cervello Hospital, University of Palermo, 90146 Palermo, Italy; 4 Medicine School, “Transilvania” University, 500036 Brasov, Romania; 5 Hospice “Casa Sperantei”, 500074 Brasov, Romania

**Keywords:** opioid, opioids, morphine, fentanyl, oxycodone, hydromorphone, dyspnea, cancer patients, systematic review, meta-analysis

## Abstract

**Background/Objectives**: Dyspnea accompanies end-of-life in many cancer patients, with around 50% experiencing moderate/severe dyspnea, and is an independent factor for poor prognosis and declining quality of life. Managing dyspnea becomes a key component of palliative treatment and end-of-life support for cancer patients. Opioids seem to be the obvious choice in cancer patients as they also address the pain component (often important in such patients). Evidence-based conclusions on the effectiveness/safety of opioids in dyspnea management are scarce, and the results are still controversial. We aim to address this knowledge gap. **Methods**: In order to achieve the objective of this paper, we conducted a comprehensive search of international databases (PubMed, Medline, Embase, and Cochrane Library) for randomized controlled trials on the use of opioids to treat refractory dyspnea in adult cancer patients, and we performed a pooled meta-analysis of the results. **Results**: The effect of opioids on the relief of dyspnea was significant (SMD −0.44, 95% CI [−0.75,−0.12], *p* = 0.007). The significance of the opioid effect is maintained only for morphine administration (SMD −078, 95% CI [−1.45,−0.10], *p* = 0.02) and only for exertional dyspnea (SMD −1.00, 95% CI [−1.98, −0.03], *p* = 0.04). No correlation was noted between fentanyl/hydromorphone and dyspnea relief or opioids administered for dyspnea at rest. The subcutaneous route seems to be significantly correlated with dyspnea relief (SMD −0.73, 95% CI [−1.27, −0.19], *p* = 0.008), while the other administration modalities lack such an effect. No significant correlation was present between the usage of morphine/fentanyl and increased odds of severe adverse effects (OR 1.48, 95% CI [0.57,3.86], *p* = 0.42); however, fentanyl seems to be associated with increased somnolence. Although we aimed to evaluate how opioids impact the quality of life of cancer patients with dyspnea, we were unable to obtain such results due to the absolute lack of the literature available discussing QoL. **Conclusions**: Although we managed to provide some insights into the efficiency and safety of opioids usage for dyspnea management in cancer patients, the evidence based on the available literature is low grade. There is a marked need to address this knowledge gap with future high-quality studies with large sample sizes and standardized protocols.

## Simple Summary

Dyspnea is frequently associated with advanced and terminal cancer, and its relief becomes a key component of symptom management in palliative care. Opioids seem to be the obvious choice for cancer patients as they also address moderate to severe pain usually also present in such patients. Evidence-based conclusions on the effectiveness and safety of opioids in dyspnea management are scarce, and the results are still controversial. We aim to address this knowledge gap by conducting a systematic review of the existing literature and a pooled meta-analysis of the results.

## 1. Introduction

Dyspnea usually accompanies the end of life in many cancer patients [1], and more than half will experience moderate to severe dyspnea [2]. This symptom is associated with worsening prognosis as an independent factor [3], and the intensity of dyspnea and the frequency of breathlessness are associated with the decline in quality of life and general condition [4,5]. Cancer patients often experience severe exertional dyspnea and sometimes even rest dyspnea, which often restricts daily life activities and social functioning of patients, leading to psychological effects (such as anger, frustration, or depression), diminished independence, and an increased need for assistance. As such, managing dyspnea becomes a key component of palliative treatment and end-of-life support for cancer patients.

Dyspnea management in cancer patients has as a prerequisite the assessment of underlying pathology and adequate treatment of all reversible causes (such as pulmonary oedema or hyperventilation due to psychologic conditions). Adjustments in daily living and activity may also contribute to dyspnea management and should be considered and implemented as a part of holistic therapeutic approaches. Environmental factors (such as allergens) also need to be considered and removed from the patient’s environment if possible. However, dyspnea in cancer patients has a non-reversible component that needs to be addressed through pharmacologic and non-pharmacologic means.

Among the existing pharmacologic means for treating the non-reversible component of cancer-related dyspnea, opioids take a center position together with benzodiazepines and are often recommended as a first-line pharmacologic intervention by several guidelines [6,7,8]. Opioids seem to be the obvious choice in cancer patients as they also address the pain component (often important in such patients). Nevertheless, evidence-based conclusions on the effectiveness and safety of opioids in dyspnea management are scarce, and the results are still controversial [9,10,11,12,13], with conclusions varying from significant improvements after opioid therapy to no effect. The available literature is also lacking when comparing opioids to other pharmacological options (for example benzodiazepines) or when comparing the effects and safety of various opioids. To the best of our ability, we could only locate three meta-analytic studies discussing opioids as therapeutic options for dyspnea management in cancer patients, but all were forced to admit inconsistent conclusions due to the low number of eligible studies and small sample size [11,12,13]. With this study, we aim to address this knowledge gap by incorporating more recently published studies. We aim at analyzing the overall effectiveness of opioids in dyspnea management in cancer patients (defined as the clinical relief of dyspnea), the effect of opioids in modifying measurable parameters (such as respiratory rate, peripheral oxygen saturation), and the security profile of opioids (adverse effects). A secondary aim of this paper is to evaluate the changes in the quality of life in cancer patients with dyspnea after opioids were administered, if sufficient data are available in the current literature. The analysis will try to compare different types of opioids and different administration routes and to compare opioids to other agents used for dyspnea management.

## 2. Materials and Methods

### 2.1. Search Strategy

In order to achieve the objective of this paper, we conducted a comprehensive review of four international databases (PubMed, Medline, Embase, and Cochrane Library) of the literature available on opioid-treated refractory dyspnea in adult cancer patients. The search was guided by the PRISMA principles for systematic reviews [14]. No limitations were imposed for publication year, and the databases were searched from inception until January 2025. All databases were searched using a combination of Boolean coordinators and relevant keywords describing the following: 1. opioids (including but not limited to: “Analgesics, Opioid” [Mesh], opioid*, opiate*, buprenorphine, codeine, fentanyl, heroin, hydrocodone, hydromorphone, laudanon, laudanum, meperidine, methadone, morphine, oxycodone, oxycontin, pentazocine, percocet, pethidine, tramadol, vicodin); 2. dyspnea (dyspnea*, dyspnoea*, dyspneic, short* of breath, breathless*); and 3. cancer patients (“Neoplasms” [Mesh], cancer*, tumor*, tumour*, neoplas*, carcinoma*, adenocarcinoma*, malignan*, oncolog*, sarcoma*). All types of opioids used for dyspnea management were considered eligible regardless of method of administration. No limitations were imposed based on cancer type. We aimed to include only high-quality research. As such, we considered optimal for inclusion only randomized controlled trials (RCT) that compared the effects of opioid usage to placebo or other pharmacologic alternatives (benzodiazepines, other opioids). All studies needed to include some form of dyspnea severity measure—either VAS (visual analog scale for dyspnea), NRS (numeric rating scale), Borg score, CPOT (clinical pain observational tool), or other such scale—as a mean of evaluating the efficacy of intervention.

Exclusion criteria included the following: 1. pediatric populations; 2. patients with other causes for dyspnea than cancer (chronic obstructive pulmonary disease—COPD, congestive heart failure); 3. non-randomized controlled trials (observational studies, single-arm studies, case reports or case series, systematic reviews, and meta-analyses); and 4. language other than English or Spanish. We considered this language limitation adequate since all databases searched provide results in English, at least as abstracts, thus making omission of potentially relevant results highly improbable. Spanish was also chosen as a possible language due to the authors’ familiarity and high number of speakers worldwide.

The primary outcome was the relief of dyspnea as a measure of efficacy of the opioid therapy. Secondary outcomes were the change in quality of life, respiratory rate, and peripheral oxygen saturation (as efficacy outcomes) as well as serious opioids adverse effects (Grade 3 or higher as per Common Terminology Criteria for Adverse Events—CTCAE) [15] and somnolence (as safety outcomes). In Table 1, we present the PICOS criteria (Population, Intervention, Comparison, Outcomes and Study framework for systematic reviews) [16] used for this research.

### 2.2. Data Extraction

The resulting papers were then processed using an automatic tool (Mendeley Reference Manager 2.94.0), and duplicate records were excluded. The remaining articles were then screened for eligibility by title and abstract by two independent authors. Only articles considered eligible by both reviewers were included in the ulterior processing. Reasons for exclusion at this phase included wrong study type or irrelevant focus. Remaining studies were then sought for full-text retrieval, and a full-text screening was performed by two independent authors assessing relevant outcomes, adequate methodology, and overall eligibility. Discrepancies were settled by a third author. Reasons for exclusions were the absence of outcomes of interest, previously published results, type of study (observational), or lack of an available English/Spanish version of the full text.

For each study, we extracted data about the authors, year of publication, sample size and types of cancers included, inclusion and exclusion criteria, study design, intervention, doses and modalities of administration, data on outcomes of interest, and summary of findings.

### 2.3. Evaluation of the Risk of Bias for Individual Studies

In order to evaluate the inherent risk of bias for each individual included study, we used the seven fields of the modified Cochrane Risk of Bias Assessment Tool [17]—two reviewers evaluated the risk of bias according to random sequence generation, allocation concealment, blinding of participants/personnel, outcome blinding and assessment, incomplete results data, selective reporting of results, and other biases. Discrepancies were then settled by consultation with one of the supervisors.

### 2.4. Statistical Analysis

All statistical analyses performed during this meta-analysis were conducted using the Review Manager 5.4 software freely available online [18]. Differences between groups and subgroups were compared using *chi*-square tests. Continuous variables were analyzed as standardized mean differences (SMDs) and 95% confidence intervals (95% CIs). Categorical variables were analyzed using odds ratios (ORs). An effect was considered significant if the OR value did not intersect 1 or if the SMD value did not cross zero. The threshold for statistical significance was considered *p*-value ≤ 0.05. We conducted a meta-analytic study of the effects of opioids when used as therapeutic options for dyspnea management in cancer patients. *I2* type statistics (which represents the proportion of total variation due to heterogeneity rather than sampling error) were used to evaluate heterogeneity between included studies, and an *I2* value >50% was considered indicative of high heterogeneity. When such highly heterogenic patterns were observed, we used random effects models in our pooled analysis. In all other cases, fixed effects models were used. Sensitivity analysis was deemed necessary only if we encountered a statistically significant result associated with an *I2* > 50%. However, no such situation occurred, so sensitivity analysis was waived. Pooled results analyzing the effectiveness of opioids in the management of dyspnea compared to placebo, additional efficacity outcomes (such as respiratory rate and peripheral oxygen saturation), and adverse effects of opioid therapy were performed and then presented as forest plots. Further subgroup analysis (by type of dyspnea, by opioid type, and by administration modality) were performed in order to discern if any discrepancies in the effectiveness of opioid appear under the influence of these factors. We also performed a multivariate analysis of the dyspnea management effect of opioids and their adverse effects taking into account the type of opioid and the type of controlled used in specific randomized controlled trials (placebo or active).

## 3. Results

### 3.1. Study Selection

**Figure 1:**
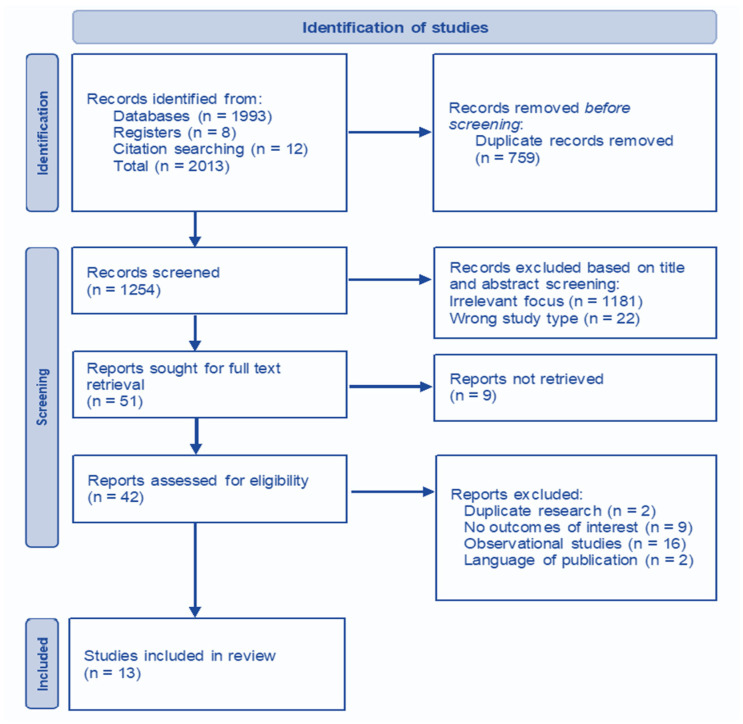
PRISMA search flow diagram.

During our databases search, we identified an initial 1993 records that seemed to meet the criteria for inclusion. An additional 20 records were identified through registers searching and manual evaluation of references. The records were processed using an open-source automated tool (Mendeley Reference Manager [19]), and 759 duplicates were removed. The remaining 1254 records were evaluated based on title and abstract, and 1181 were excluded for irrelevant focus and 22 for not being the correct study type (non-RCT). Out of the 51 studies sought for full-text retrieval, nine were unavailable. The final 42 records were furthered screened, and 29 were excluded (duplicate research, no outcomes of interest, wrong study type, and not available in English/Spanish). The final 13 RCTs were included in this analytic study. There were a few promising RCTs that seemed to meet the inclusion criteria but finally were rejected due to including mixed populations with a high number of benign disease patients that may interfere with the analysis [20], comparing the effects of opioids to non-pharmacologic methods such as acupuncture [21], or not available in English or Spanish [22]. The PRISMA search flow diagram is presented in Figure 1.

### 3.2. Risk of Bias

**Figure 2:**
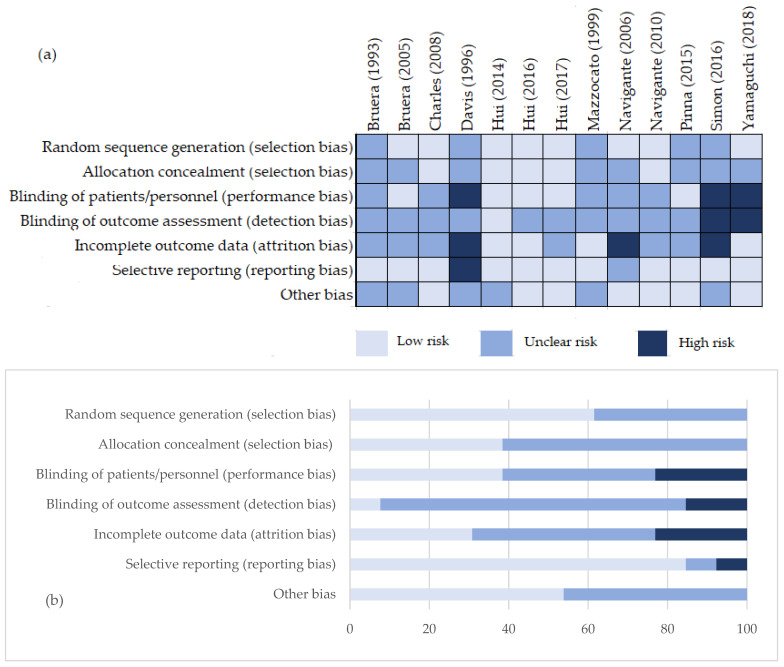
Risk of bias assessment. (**a**) Authors’ judgement about each risk of bias item for each included study [23,24,25,26,27,28,29,30,31,32,33,34,35]; (**b**) authors’ judgements about each risk of bias item presented as percentages across all included studies.

The inherent risk of bias for each individual included study was evaluated using the modified Cochrane Risk of Bias Assessment Tool. The author’s judgement is presented in Figure 2 both as results for each study and as an overall risk across studies. Three studies were evaluated as low risk [23,24,25], four as high risk [26,27,28,29], and six as intermediate (unclear) risk [30,31,32,33,34,35].

### 3.3. Characteristics of Included Studies

A total of 397 patients were enrolled in the 13 randomized controlled trials included. Sample sized had a median of 30.5 enrolled patients (varying from 9 to 101). All studies included cancer patients with rest/exertional dyspnea. Most patients had primary lung cancer or lung metastases, but the study cohort also included other forms of cancers such as gastro-intestinal, genito-urinary, sarcomas, or hematologic cancers. Placebo control was used in eight studies [23,24,25,28,30,32,33,35], while active control was used in five [26,27,29,31,34]. In three studies, opioids were compared to other opioids [26,27,31] and with benzodiazepines in two studies [29,34]. Opioids were administered through subcutaneous injections, transmucosal path, per oral, and in nebulized form. To the best of our ability, we could not identify any studies describing the effect of intravenous opioids. The main characteristics of the included studies are presented in Table 2.

### 3.4. Primary Outcome—Dyspnea Relief

**Figure 3:**
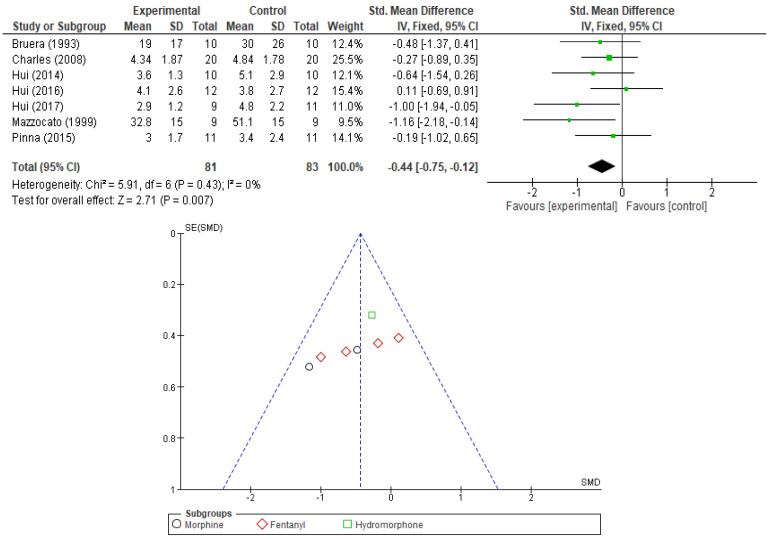
Forest plot comparing the effect of opioids vs. PL in relief of dyspnea and corresponding funnel plot (showing lack of publication bias) [23,24,25,30,32,33,35].

The primary outcome was evaluated in seven studies with morphine, five with fentanyl, one with oxycodone (this study is also counted as a Fentanyl study since it compares the effects of the two opioids), and one with hydromorphone. From these studies only those that expressed the intensity of dyspnea as a continuous variable or for which a clearance interval could be estimated and did not compare various forms of opioids were introduced in the pooled analysis. Finally, seven RCTs—two with morphine, four with fentanyl, and one with hydromorphone—were used for the integrated analysis, all of which were placebo controlled. The effect of opioids was significant (SMD −0.44 95% CI [−0.75,−0.12], *p* = 0.007) (Figure 3). No sensitivity analysis was performed for this outcome due to the absence of heterogeneity between studies (*I2* = 0%). No significant publication bias was observed for this outcome when examining the corresponding funnel plot.

To better discern the intricacies of the opioid effect on the primary outcome (dyspnea relief) we performed additional subgroup analysis evaluating the results by opioid type, administration modality, and type of dyspnea. In Table 3, we summarize these findings highlighting in bold the results that maintain statistical significance and a low level of heterogeneity. The significance of the opioid effect is maintained only for morphine administration (SMD −078, 95% CI [−1.45,−0.10], *p*-value = 0.02) and only for exertional dyspnea (SMD −1.00, 95% CI [−1.98, −0.03], *p*-value = 0.04). No correlation was noted between fentanyl or hydromorphone and dyspnea relief. Opioids seem to play a role in the relief of exertional dyspnea but not dyspnea at rest. Also, the subcutaneous administration seems to be significantly correlated with dyspnea relief (SMD −0.73, 95% CI [−1.27, −0.19], *p*-value = 0.008), while the other administration modalities lack such effect.

**Figure 4:**
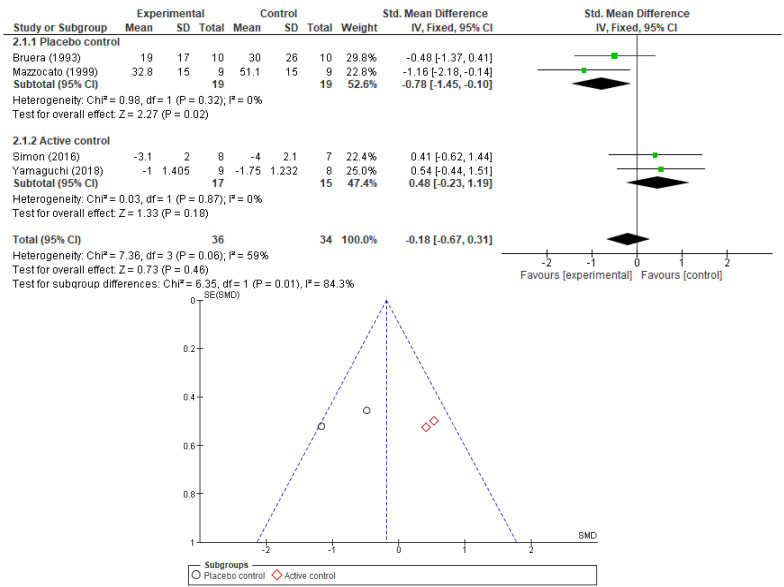
Forest plot comparing the effect of morphine on dyspnea relief: subgroup analysis by type of control used in RCT (placebo or active control studies) and corresponding funnel plot (showing lack publication bias) [26,27,30,33].

**Figure 5:**
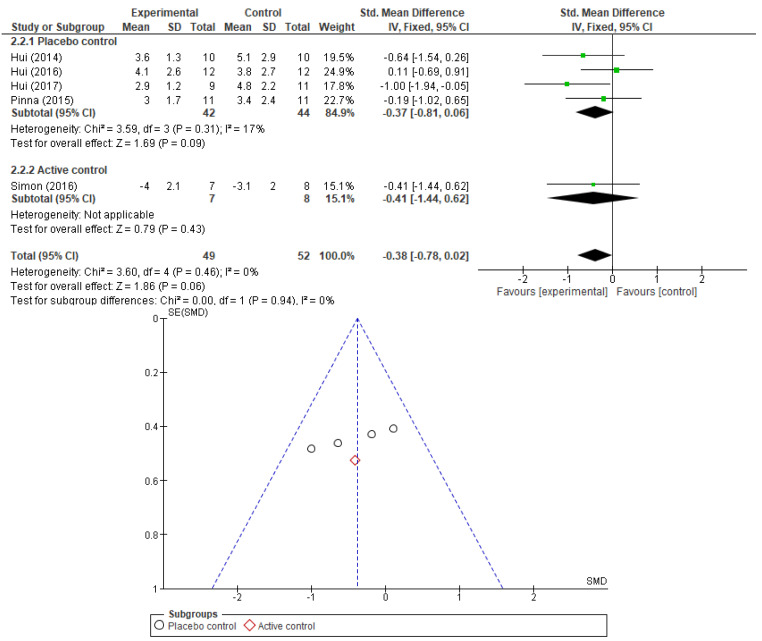
Forest plot comparing the effect of fentanyl in relief of dyspnea: subgroup analysis by type of control used in RCT (placebo or active control studies) and corresponding funnel plot (showing lack of publication bias) [23,24,25,26,35].

Additionally, we wanted to find out if the type of control used in the RCTs has any influence on the perceive effect of opioids in dyspnea relief. We compared the results of trials using placebo as control and those using active controls. The results are presented as forest plots in Figure 4 and Figure 5 (for morphine and fentanyl). Only when compared to placebo controls, the morphine effect maintained its significance—SMD −0.78, 95% CI [−1.45, −0.10], *p*-value = 0.02, *I2* = 0%. When compared to active controls the significance was lost—SMD 0.48, 95% CI [−0.23, 1.19], *p*-value = 0.18, *I2* = 0%. The difference in effect between the two subgroups was significant (*p*-value = 0.01). For fentanyl, we could not establish any significant correlation with dyspnea relief despite the type of control used (placebo—SMD −0.37, 95% CI [−0.81, 0.06], *p*-value = 0.09; active control—SMD −0.41, 95% CI [−1.44, 0.62], *p*-value = 0.43), and this result is in concordance with the previous subgroup analysis.

### 3.5. Secondary Outcomes

Secondary outcomes included three efficacy indicators (quality of life, respiratory rate, and peripheral oxygen saturation) and two safety indicators (reported severe adverse reactions and somnolence).

No RCT discussing the quality of life (QoL) in cancer patients receiving opioids for dyspnea management met the inclusion criteria in the current meta-analysis. We could only identify one study [21] that approached the subject of QoL after opioid therapy; however, this study compared the effects of morphine to a non-pharmacologic alternative (acupuncture). Therefore, it was considered unsuitable for analysis.

**Figure 6:**
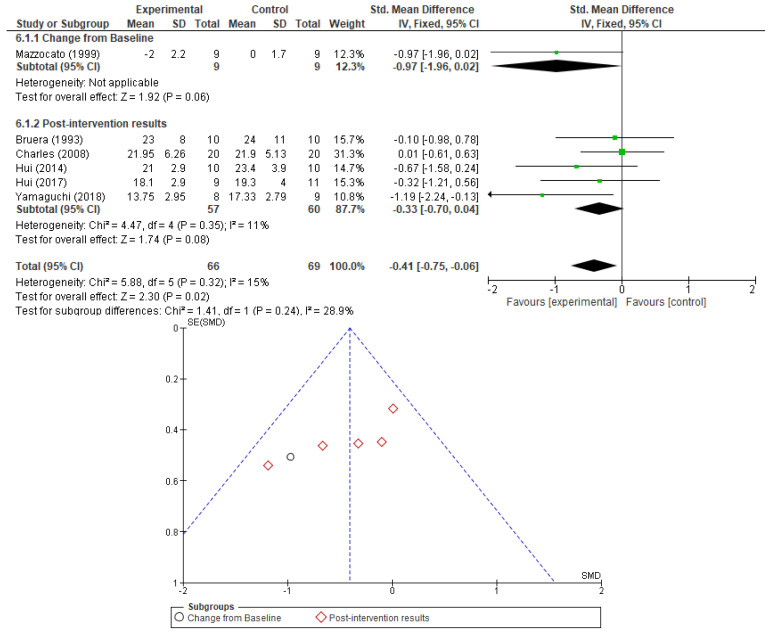
Respiratory rate analysis as a measure of opioid effect and the corresponding funnel plot [23,24,27,30,32,33].

**Figure 7:**
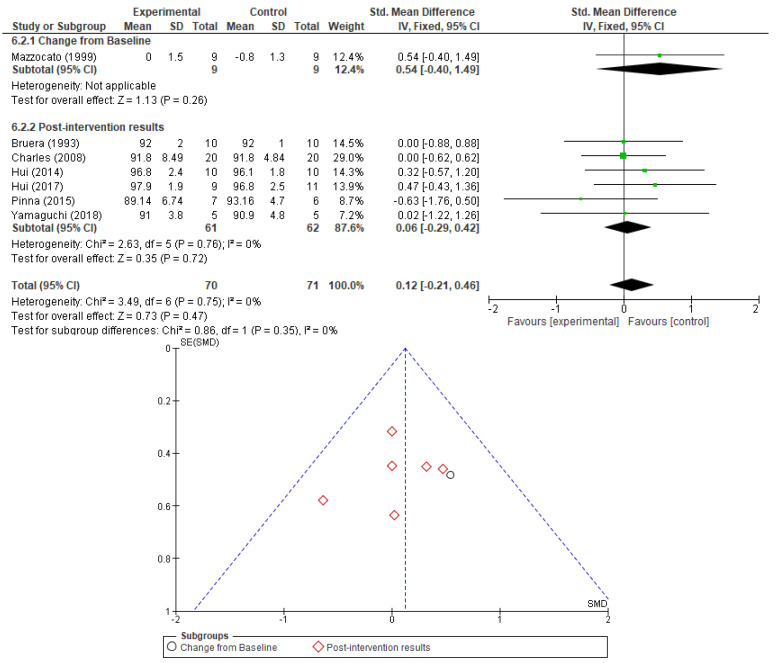
Peripheral oxygen saturation analysis as a measure of opioid effect and the corresponding funnel plot [23,24,27,30,31,33,35].

Information on respiratory rate (RR) and peripheral oxygen saturation (SaO_2_) was included in six and seven RCTs, respectively. No correlation could be established between opioid therapy and a detrimental effect on respiratory rate or a corresponding improvement in peripheral oxygen saturation, neither as changes from baseline levels nor as post-intervention results. Results are presented in Figure 6 and Figure 7. No sensitivity analysis was performed due to low heterogeneity. No significant publication bias was found when investigating the corresponding funnel plots.

**Figure 8:**
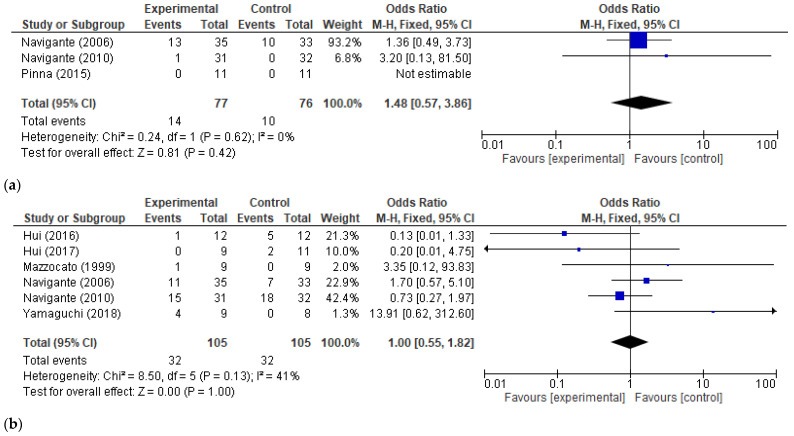
Opioid treatment-related adverse reactions. (**a**) Forest plot for severe adverse effects (SAEs) caused by opioids vs. control—overall effect; (**b**) Forest plots for somnolence caused by opioids vs. control—overall effect [23,25,27,29,33,34,35].

**Figure 9:**
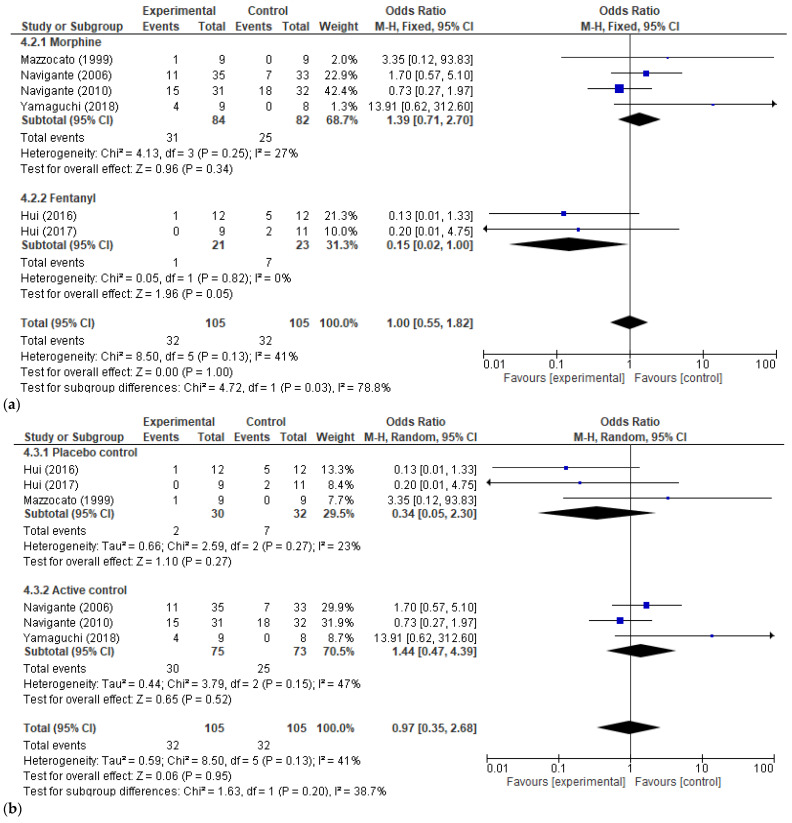
Subgroup analysis of opioid treatment-related somnolence. (**a**) By type of opioid; (**b**) by type of control—placebo vs. active control [23,25,27,29,33,34].

The safety secondary outcomes we analyzed were the severe adverse reactions related to opioid usage in general and somnolence in particular (as the most frequent complication of therapy). Three studies [29,34,35] presented information on severe adverse reactions and 6 [23,25,27,29,33,34] offered details on somnolence. No significant correlation was present between the usage of morphine/fentanyl for dyspnea relief in cancer patients and increased odds of treatment-related severe adverse effects or somnolence (OR 1.48, 95% CI [0.57, 3.86], *p*-value = 0.42 and OR 1.00, 95% CI [0.55, 1.82], *p*-value = 1.00, respectively). Although there was no high heterogeneity observed between studies (*I2* = 41%) for the somnolence outcome, we could perform a subgroup analysis to see if the type of opioid used and the type of study control may influence the overall effect. When analyzing somnolence by opioid type, we encountered a significant subgroup difference (*I2* = 78.8%, *p*-value = 0.03). Fentanyl usage was associated with increased somnolence odds (OR 0.15, 95% CI [0.02–1.00]), and this was significant (*p*-value = 0.05, *I2* = 0%). No such association occurred when analyzing morphine usage (OR 1.39, 95% CI [0.71, 2.70], *p*-value = 0.34). The odds of somnolence occurring was not influenced in any way by the type of control used in the RCTs (placebo or active). These results are presented in Figure 8 and Figure 9.

## 4. Discussion

In our study, we included 13 RCTs with a total of 397 patients. The majority of studies offered information on Morphine and fentanyl; however, for all other opioids, there is a scarcity of literature (oxycodone and hydromorphone effects were described in one study each, and no additional studies on other type of opioids could be located, thus making it impossible to assess the effects and safety of these opioids). Unfortunately, the quality of the included studies is not optimal due to small sample sizes (some of them with less than 10 patients per arm) and unclear/high risk of bias. Additionally, methodological shortcomings may have influenced the results. The patients enrolled belong to an especially frail category (as exemplified by an average time until death of 5 days reported by some studies [36]), thus making it, by definition, unethical to deny crossover from placebo to active treatment. Crossover, although understandable in these situations, gives rise to increased risk of cofounding and difficult analysis of results. Concerns about patients’ safety and comfort may also justify the shorter washout periods observed in some studies [12,30,33] when patients crossover. The fact that cancer patients with symptomatic dyspnea may deteriorate suddenly and irreversible is a reality that makes it extremely difficult to conduct large randomized double-blind controlled trials with rigorous protocols, thus making it acceptable to include such a study in a pooled meta-analysis study with the current level of evidence. Another barrier in conducting high-quality studies analyzing the effects of opioids in cancer-related dyspnea is the “opiophobia”—the concern that these drugs may cause respiratory depression and hasten death [37]—which may limit enrolment. In addition to previously discussed limitations, we have to remark that there is a high variability between how the results are reported—in some studies, we could not extrapolate usable data for the integrated studies although the studies discussed the outcomes of interest. Although standardized, the forms used to evaluate dyspnea relief across studies vary widely (VAS, NRS, Borg score, 6MWT), thus limiting the possibilities of analysis.

Even so, we were able to draw some usable conclusions that add to the existing knowledge. The primary outcome analysis (dyspnea relief) showed the overall superiority of opioids when compared to placebo. However, the superiority was not maintained when comparing the opioids to active controls. This leaves room for the possible inferiority of results to other pharmacologic options (such as benzodiazepines); however, such conclusions cannot be drawn at this time due to the lack of relevant studies and small sample sizes. Our findings are broadly concordant with the previous meta-analysis by Takagi et al. [38], which also suggested modest benefit of opioids for dyspnea relief in cancer patients. Similarities in pooled estimates likely reflect the limited and largely overlapping body of randomized evidence available in this field. However, our analysis incorporated an updated search period, distinct eligibility criteria, and additional subgroup analyses focusing on dyspnea phenotype, administration route, and comparator type. The subgroup analysis also seemed to indicate that the observed superiority maintains significance only when administering morphine (no correlation was noted between fentanyl or hydromorphone and dyspnea relief) and only when the opioids are used for exertional dyspnea relief (no significant correlation was found between opioid usage and rest dyspnea relief). Several potential reasons contribute to the limited effectiveness of opioids other than morphine in the management of dyspnea in cancer patients. One factor is the pharmacokinetic variability among opioids. Morphine has a relatively predictable onset and duration of action, which facilitates titration and monitoring. Other opioids, such as fentanyl and oxycodone, have variable absorption rates depending on the route of administration and patient-specific factors, such as hepatic or renal function, which may lead to inconsistent symptom control. The route of administration also plays a role. Morphine is often given orally or subcutaneously, both effective for managing dyspnea in palliative settings. Other opioids, particularly fentanyl, are commonly administered via transdermal or intranasal routes, which may not provide the rapid relief required for acute dyspnea episodes. This may limit their utility in urgent symptom management. We have to mention that no real possibility of evaluating opioids such as oxycodone or methadone was available (no relevant studies). Also, most of the studies on fentanyl focused on exertional dyspnea, and this choice can influence the results of the current analysis. Subcutaneous administration seems to be significantly correlated with dyspnea relief, while the other administration modalities lack such effect.

QoL is the most important measurement of the efficacity of therapeutic and lifestyle interventions and provides prognostic information on patient survival and subsequent evolution [39]. No RCT discussing the QoL of cancer patients receiving opioids for dyspnea management met the inclusion criteria in the current meta-analysis. We could only identify one study [21] that approached the subject of QoL after opioid therapy. However, this study compared the effects of morphine to a non-pharmacologic alternative (acupuncture); therefore, it was considered unsuitable for analysis.

No correlation could be established between opioid therapy and a detrimental effect on respiratory rate or a corresponding improvement in peripheral oxygen saturation, neither as changes from baseline levels nor as post-intervention results. This is partially contrary to previous studies that showed that opioids may induce a decline in respiratory rate [40]. As such, the evidence on the safety of opioid usage in cancer-related dyspnea remains controversial.

The safety secondary outcomes we analyzed were the severe adverse reactions related to opioid usage in general and somnolence in particular (as the most frequent complication of therapy). No significant correlation was present between the usage of morphine/fentanyl for dyspnea relief in cancer patients and increased overall odds of treatment-related severe adverse effects or somnolence. However, only fentanyl seemed to increase somnolence.

### Study Limitations

Conducting proper randomized controlled studies in palliative care, especially when such involving cancer patients (who are very vulnerable, prone to rapid irreversible decline, and present unique challenges in obtaining consent and psychological issues) is more difficult than in other medical fields [41,42]. Unfortunately, the number of studies included in this meta-analysis is small, and most sample sizes were very limited. A high percentage of the included studies were considered high risk of bias (30%) providing results with a low level of evidence. In addition, many studies allowed for crossover but had a very short period of washout, which may lead to potential biases in outcome assessment. We found it difficult to analyze the safety of the opioid intervention due to the limited number of studies reporting comprehensively on adverse effects. In addition, there is little to no literature available evaluating QoL outcomes in cancer patients receiving opioid therapy for dyspnea management or evaluating the effect of some opioids like oxycodone or hydromorphone. Similar to problems noted for cancer survivorship or choosing a tailored therapeutic approach for each patient [43,44,45,46], palliative care also faces its challenges, and thus far, adequate studies for clarifying the effect and safety of the intervention are lacking. Another potential source of bias is the exclusion of studies published in other languages than English and Spanish, and this may result in potentially omitting relevant results of the database search.

## 5. Conclusions

Although we managed to provide some insights into the efficiency and safety of opioid usage for dyspnea management in cancer patients (1. Morphine seems to be the only type of opioid significantly associated with dyspnea relief; 2. Opioids seem to be effective only in the relief of exertional dyspnea and to a lesser degree for rest dyspnea; 3. The subcutaneous route of administration seems to be more effective in delivering optimal opioid dyspnea management; and 4. Opioids seem not to increase the frequency of severe adverse reactions, but fentanyl is associated with increased somnolence), the evidence based on the available literature is low grade. There is a marked need to address this knowledge gap using future high-quality studies with large sample sizes and standardized protocols and scales for measuring the effects of the intervention. Such studies should address both the efficiency and safety of opioids (including less used opioids such as oxycodone or hydromorphone). QoL in post-intervention settings should also be clarified through more rigorous research.

## Figures and Tables

**Table 1:** PICOS criteria for inclusion of trials.

Parameter	Inclusion Criteria
Participants	Adult cancer patients with refractory dyspnea
Intervention	Opioids administered subcutaneous, intravenous, per oral, or transmucosal for dyspnea
Comparison	Pharmacologic alternatives for dyspnea management—placebo or active control (benzodiazepines, other opioids)
Outcomes	Relief of dyspnea, intensity of dyspnea Quality of life Respiratory rate, peripheral oxygen saturation Severe adverse effects, somnolence
Study design	Randomized controlled trials

**Table 2:** Characteristics of included RCT.

Study	Intervention	Sample Size	Study Design	Inclusion Criteria	Exclusion Criteria	Outcomes				
						Dyspnea	Somnolence	SAE	Additional Outcomes	Summary of Findings
Bruera (1993) [30]	Mo sc vs. PL 34 ± 12 mg Mo Duration of intervention—48 h Washout period—24 h	10	RCT, crossover, PL controlled	Progressive disease, conscious patients, normal cognition, rest dyspnea, receiving continuous oxygen 2 to 6 L/min, no information on location of patients (ICU/ward/hospice/home care)	NS	+	-	-	RR SaO_2_	Mo administration was superior to PL (significantly lower VAS at 30, 45, 60 min after intervention). Intermittent Mo is safe and effective for the management of dyspnea in terminally ill cancer patients.
Bruera (2005) [31]	Mo sc vs. Mo inhal Equivalent dosage as previous opioid used Same dose in both sc and nebulized phase	11	RTC, crossover, double-blind, active controlled	Rest cancer-related dyspnea, NRS ≥ 3/10 regular oral/parenteral opioids with no dose change for 72 h; normal cognition status; no information on location of patients (ICU/ward/hospice/home care)	Mo contraindications; Acute dyspnea due to pneumonia, embolism or congestive heart failure	+	+	+	NS	No significant changes in NRS at 60 min after intervention from baseline conditions
Charles (2008) [32]	5 mg nebulized/ systemic HiMo vs. 3 mg nebulized PL Duration of intervention—72 h	20	RCT, crossover, double-blind	Inpatient and community-based patients, >18 yo, diagnosis of cancer with a clinical prognosis of at least seven days, Mini-Mental State Examination scores > 24/30, incident dyspnea with non-reversible components on a background of either irreversible dyspnea at rest or development of dyspnea when they spoke	NS	+	-	-	RR SaO_2_	Dyspnea decreased significantly for all treatments, with no significant difference in NRS change after intervention from baseline conditions. Only nebulized HiMo produced a rapid clinically significant improvement in dyspnea.
Davis (1996) [28]	Nebulized Mo vs. PL 5–50 mg Mo vs. 5 mL saline Duration of intervention—2 days	79	RCT, double-blind, crossover and stratified, PL controlled	Adult patients with cancer with rest dyspnea and Rx. evidence of pleural effusion, lymphadenopathy, lymphangitis, carcinomatosis, or mass lesion(s); no information on location of patients (ICU/ward/hospice/home care)	Opioid intolerance Poor renal function	+	-	-	NS	The results do not support the use of nebulized Mo for dyspnea in cancer patients (no significant VAS change)
Hui (2014) [24]	Fent sc vs. PL 30–350 mcg Fent equivalent to 15–25% of Mo MEDD vs. saline	20	RCT, double-blind, parallel, PL controlled	Ambulatory ≥ 18 yo patients with breakthrough dyspnea NRS ≥ 3/10, KPS score ≥ 50%, MEDD between 30–580 mg/day	NRS ≥ 7/10, O_2_ need >6 L/min, delirium, allergy, substance abuse, coronary artery disease, tachycardia, or HTA	+	-	-	6MWT Borg score RR SaO_2_	No proper subgroup analysis, but a trend toward improvement of exertion dyspnea NRS after Fent sc was noted. Prophylactic Fent seems to be and improves dyspnea, fatigue, walk distance, and RR.
Hui (2016) [23]	Fent tm vs. PL 100–400 mcg nebulized Fent pectin nasal spray (FPNS) equivalent to 15–25% of Mo MEDD vs. PL	24	RCT, double-blind, parallel, PL controlled	Ambulatory ≥ 18 yo patients with breakthrough dyspnea NRS ≥ 3/10, KPS ≥ 50%, MEDD between 80–580 mg/day	NRS ≥ 7/10, O_2_ need > 6 L/min, delirium, allergy, opioid abuse, unable to complete 6MWT	+	+	-	6MWT	No proper subgroup analysis, but a trend toward improvement of NRS after Fent tm at 20 min after intervention was noted. FPNS seemed safe, reduced rest dyspnea, and increased walking distance. However, the PL effect was also substantial.
Hui (2017) [25]	Fent tm vs. PL 100–200 mcg oral fentanyl buccal tablet (FBT) equivalent to 20–50% Mo MEDD vs. PL	20	RCT, double-blind, parallel, PL controlled	Ambulatory ≥ 18 yo patients with breakthrough dyspnea NRS ≥ 3/10, KPS ≥ 50%, MEDD between 60–130 mg/d	NRS ≥ 7/10, O_2_ need > 6 L/min, delirium, allergy, opioid abuse, unable to complete 6MWT	+	+	-	6MWT Borg score RR SaO_2_	No significant difference between groups in NRS after a 6-min walk, but prophylactic FBT may offer a reduction of exertional dyspnea and is well-tolerated.
Mazzocato (1999) [33]	Mo sc vs. PL 5 mg Mo Duration of intervention—48 h Washout period—24 h	9	RCT, crossover, double-blind, PL controlled	Patients with cancer-related dyspnea, in the absence of brain tumors or acute respiratory decompensation; no information on location of patients (ICU/ward/hospice/home care)	NS	+	+	-	Borg score RR SaO_2_	Mo administration was superior to PL (significant greater change of VAS at 45 min after intervention); Mo seems to be effective for cancer-related dyspnea management.
Navigante (2006) [29]	Mo sc vs. midazolam sc vs. Mo + midazolam	101	RCT, single-blinded, active controlled	Patients with terminal cancer ≥ 18 yo, with severe rest dyspnea, life expectancy of <1 month, Mini-Mental Status Exam > 23/30, ECOG = 4; no information on location of patients (ICU/ward/hospice/home care)	COPD Renal/hepatic failure Congestive heart failure Uncontrolled symptoms	+	+	+	Borg score	No significant difference in modified Borg score between groups at 24 and 48 h after interventions, but data seemed to suggest that the addition of midazolam to Mo may have beneficial effects in controlling baseline levels of dyspnea.
Navigante (2010) [34]	Mo po vs. midazolam po 3 mg Mo vs. 2 mg midazolam, with an incremental step of 25% until reaching effective dose in both arms Duration of intervention—5 days	63	RCT, single-blind, parallel, active controlled	Ambulatory patients, ≥18 yo, Mini-Mental Status Examination score > 23/30, moderate/severe rest dyspnea	COPD Severe renal/ hepatic failure NRS ≥ 3/10 SaO_2_ < 85%	+	+	+	Adverse events	Midazolam administration was associated with better NRS and appeared to be the better option for the immediate and long-term relief of dyspnea
Pinna (2015) [35]	Fent tm vs. PL 200–400 ug oral transmucosal fentanyl citrate (OTFC) vs. PL Duration of intervention: Noted as “depended on the study design” in the study. Washout period: >48 h	13	RCT, double blind, crossover, PL controlled	Cancer patients in home care with moderate effort dyspnea, KPS ≥ 50%, hemoglobin >10 mg/dL, SaO_2_ > 90%	COPD	+	-	+	6MWT SaO_2_	No significant difference in NRS between groups at any time measured A significant PL effect was observed in all the patients
Simon (2016) [26]	Mo po vs. Fent tm Oral immediate-release morphine 1/6 DOME vs. 100–600 ug oral Fent buccal tablet Duration of intervention: 10 days; washout period: 24 h	10	RCT, multicenter, open-label, active-controlled, crossover	≥18 yo, NRS ≥ 3/10, opioid tolerant, no information on location of patients (ICU/ward/hospice/home care)	Uncontrolled dyspnea, severe renal/hepatic impairment, opioid abuse	+	-	-	RR SaO_2_	No significant difference in NRS change from baseline between groups at 10 and 30 min after intervention but a faster and greater relief of episodic was observed with Fent; no safety concerns in any arm.
Yamaguchi (2018) [27]	Mo po vs. Oxy po 18.89 ± 15.23 mg Mo vs. 5.31 ± 4.91 mg Oxy	17	RCT, multicenter, open-label, parallel-group	Cancer patients in palliative care units, ≥20 yo, with malignant disease; regular use of Oxy; moderate/ severe rest dyspnea; SaO_2_ > 90%; no cognitive impairment	Hg < 7 g/dL; organ dysfunction; uncontrolled pain; life expectancy of <1 month, allergy, bacterial colitis, rescue opioids < 9 h prior; regular opioids use other than Oxy	+	+	-	RR SaO_2_	No significant difference in NRS change from baseline between groups at 60 and 120 min after intervention. The study failed to prove the non-inferiority of Oxy when compared to Mo, and Oxy may be effective and safe for cancer-related dyspnea management.

RCTs—randomized controlled trials; n—sample size (number of patients included); SAEs—severe adverse reactions; NS—not specified; HTA—arterial hypertension; RR—respiratory rate; SaO_2_—peripheral oxygen saturation; VAS—visual analog scale for dyspnea; ECOG—Eastern Cooperative Oncology Group; NRS—numerical rating scale; Mo—morphine; Oxy—oxycodone; Fent—Fentanyl; HiMo—hydromorphone; PL—placebo; po—oral administration; sc—subcutaneous administration; tm—transmucosal administration; inhal—inhalation; KPS—Karnofsky Performance Status; MEDD—Morphine Equivalent Daily Dose; COPD—Chronic Obstructive Pulmonary Disease; DOME—daily oral morphine equivalent dose; Effective dose—Dose that reduced the intensity of dyspnea by at least 50%; 6MWT—6-min walk test; yo—years old.

**Table 3:** Subgroup analysis for primary outcome (dyspnea relief).

Subgroup Analysis	Variable for Subgroup Analysis	Nr. of Studies	Nr. of Patients Opioids/Control	*I* ^2^	SMD [95% CI]	*p*-Value
Opioid type	**Morphine**	**2**	**19/19**	**0%**	**−** **0.78 [−1.45, −0.10]**	**0.02**
	Fentanyl	4	42/44	17%	−0.37 [−0.81, 0.06]	0.09
	Hydromorphone	1	20/20	NA	−0.27 [−0.89, 0.35]	0.4
Administration modality	**Subcutaneous**	**3**	**29/29**	**0%**	**−** **0.73 [−1.27, −0.19]**	**0.008**
	Nebulized/Inhalation	1	20/20	NA	−0.27 [−0.89, 0.35]	0.4
	Transmucosal	3	32/34	37%	−0.29 [−0.79, 0.20]	0.24
Type of dyspnea	Rest dyspnea	3	39/39	73%	−8.28 [−21.03, 4.47]	0.2
	**Exertional dyspnea**	**4**	**42/34**	**14%**	**−** **1.00 [−1.98, −0.03]**	**0.04**

SMD—standard mean difference (calculated for continuous variables using inverse variance method and fixed/ random effects models according to low/high heterogeneity); NA—not applicable; 95% CI—95% confidence interval.

## Data Availability

All studies included in this review are available online.

## Abbreviations

The following abbreviations are used in this manuscript:

COPD	Chronic obstructive pulmonary disease
CTCAE	Common Terminology Criteria for Adverse Events
PICOS	Population, Intervention, Comparison, Outcomes and Study framework for systematic reviews
SMD	Standardized mean differences
OR	Odds ratio
95% CI	95 percent confidence interval
RCTs	Randomized controlled trials
Mo	Morphine
Fent	Fentanyl
HyMo	Hydromorphone
Oxy	Oxycodone
po	Per oral
sc	Subcutaneous administration
tm	Transmucosal administration
Inhal	Inhalation
SAEs	Severe adverse reactions
NS	Not specified
HTA	Arterial hypertension
RR	Respiratory rate
SaO_2_	Peripheral oxygen saturation
VAS	Visual analog scale for dyspnea
CPOT	Clinical pain observational tool
ECOG	Eastern Cooperative Oncology Group
NRS	Numeric rating scale
QoL	Quality of life
KPS	Karnofsky performance status
MEDD	Morphine equivalent daily dose
DOME	Daily oral morphine equivalent dose. Effective dose: Dose that reduced the intensity of dyspnea by at least 50%.
6MWT	6-min walk test
OTFC	Oral transmucosal fentanyl citrate
FBT	Fentanyl buccal tablet
FPNS	Fentanyl pectin nasal spray
