# Correction: Chitoran et al. A Systematic Review and Meta-Analysis on Opioid Management of Dyspnea in Cancer Patients. *Cancers* 2025, *17*, 1368

**DOI:** 10.3390/cancers18091488

**Published:** 2026-05-06

**Authors:** Elena Chitoran, Vlad Rotaru, Giuseppe Gullo, Daniela Viorica Mosoiu, Laurentiu Simion

**Affiliations:** 1 Medicine School, “Carol Davila” University of Medicine and Pharmacy, 050474 Bucharest, Romania; 2 General Surgery and Surgical Oncology Department I, Bucharest Institute of Oncology “Prof. Dr. Alexandru Trestioreanu”, 022328 Bucharest, Romania; 3 Department of Obstetrics and Gynecology, Villa Sofia Cervello Hospital, University of Palermo, 90146 Palermo, Italy; 4 Medicine School, “Transilvania” University, 500036 Brasov, Romania; 5 Hospice “Casa Sperantei”, 500074 Brasov, Romania

## Missing Citation

Following publication of this article [1], the authors wish to acknowledge a relevant previously published meta-analysis that was not cited in the original manuscript: Takagi, Y.; Sato, J.; Yamamoto, Y.; Matsunuma, R.; Watanabe, H.; Mori, M.; Hasegawa, T.; Matsuda, Y.; Kako, J.; Kasahara, Y.; et al. Opioids for the Management of Dyspnea in Cancer Patients: A Systematic Review and Meta-Analysis. *Int. J. Clin. Oncol.* **2023**, *28*, 999–1010. https://doi.org/10.1007/S10147-023-02362-6.

The omission of this citation was unintentional. The authors recognize that this prior meta-analysis addressed a closely related clinical question and should have been referenced in the Discussion sections to better contextualize the current evidence base.

The citation has now been inserted in Discussion, Paragraph Number 2 and should read:

Even so, we were able to draw some usable conclusions that add to the existing knowledge. The primary outcome analysis (dyspnea relief) showed the overall superiority of opioids when compared to placebo. However, the superiority was not maintained when comparing the opioids to active controls. This leaves room for the possible inferiority of results to other pharmacologic options (such as benzodiazepines); however, such conclusions cannot be drawn at this time due to the lack of relevant studies and small sample sizes. Our findings are broadly concordant with the previous meta-analysis by Takagi et al. [38], which also suggested modest benefit of opioids for dyspnea relief in cancer patients. Similarities in pooled estimates likely reflect the limited and largely overlapping body of randomized evidence available in this field. However, our analysis incorporated an updated search period, distinct eligibility criteria, and additional subgroup analyses focusing on dyspnea phenotype, administration route, and comparator type. The subgroup analysis also seemed to indicate that the observed superiority maintains significance only when administering morphine (no correlation was noted between fentanyl or hydromorphone and dyspnea relief) and only when the opioids are used for exertional dyspnea relief (no significant correlation was found between opioid usage and rest dyspnea relief). Several potential reasons contribute to the limited effectiveness of opioids other than morphine in the management of dyspnea in cancer patients. One factor is the pharmacokinetic variability among opioids. Morphine has a relatively predictable onset and duration of action, which facilitates titration and monitoring. Other opioids, such as fentanyl and oxycodone, have variable absorption rates depending on the route of administration and patient-specific factors, such as hepatic or renal function, which may lead to inconsistent symptom control. The route of administration also plays a role. Morphine is often given orally or subcutaneously, both effective for managing dyspnea in palliative settings. Other opioids, particularly fentanyl, are commonly administered via transdermal or intranasal routes, which may not provide the rapid relief required for acute dyspnea episodes. This may limit their utility in urgent symptom management. We have to mention that no real possibility of evaluating opioids such as oxycodone or methadone was available (no relevant studies). Also, most of the studies on fentanyl focused on exertional dyspnea, and this choice can influence the results of the current analysis. Subcutaneous administration seems to be significantly correlated with dyspnea relief, while the other administration modalities lack such effect.

With this correction, the order of some references has been adjusted accordingly. The authors apologize for any inconvenience caused and state that the scientific conclusions are unaffected. This correction was approved by the Academic Editor. The original publication has also been updated.
